# Optical signatures of single ion tracks in ZnO†

**DOI:** 10.1039/c9na00677j

**Published:** 2019-12-23

**Authors:** G. C. Vásquez, K. M. Johansen, A. Galeckas, L. Vines, B. G. Svensson

**Affiliations:** Centre for Materials Science and Nanotechnology, University of Oslo N-0318 Oslo Norway g.c.vasquez@smn.uio.no

## Abstract

The optical properties of single ion tracks have been studied in ZnO implanted with Ge by combining depth-resolved hyperspectral cathodoluminescence (CL) and photoluminescence (PL) spectroscopy techniques. The results indicate that ZnO is susceptible to implantation doses as low as 10^8^ to 10^9^ cm^−2^. We demonstrate that the intensity ratio of ionized and neutral donor bound exciton emissions [D^+^X/D^0^X] can be used as a tracer for a local band bending both at the surface as well as in the crystal bulk along the ion tracks. The hyperspectral CL imaging performed at 80 K with 50 nm resolution over the regions with single ion tracks permitted direct assessment of the minority carrier diffusion length. The radii of distortion and space charge surrounding single ion tracks were estimated from the 2D distributions of defect-related green emission (GE) and excitonic D^+^X emission, both normalized with regard to neutral D^0^X emission, *i.e.*, from the [GE/D^0^X] and [D^+^X/D^0^X] ratio maps. Our results indicate that single ion tracks in ZnO can be resolved up to ion doses of the order of 5 × 10^9^ cm^−2^, in which defect aggregation along the extended defects obstructs signatures of individual tracks.

## Abstract 2

This paper shows a methodology to identify optical signatures from single ion tracks as an approach to study locally the defects created during ion implantation.

## Graphical abstract

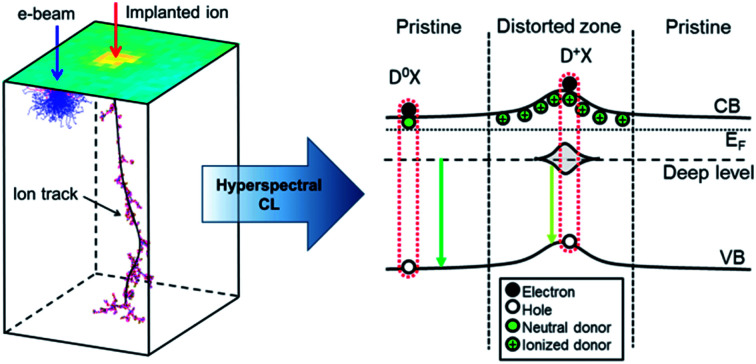

## Introduction

Over the past decades, ion implantation has been used as a technique to both precisely engineer and study fundamental properties of semiconductors.^1,2^ In the process, the ion projectiles are accelerated towards and penetrate the target material. The energetic ions lose their kinetic energy by electronic and nuclear interaction with the host material, creating point defects and lattice distortions along the trajectory, so called ion-tracks. Ion implantation allows for precise control of the ion energy and dose, so by adjusting the implantation and subsequent annealing conditions, the cumulative effect of ion implantation can be tuned to modify the electrical, mechanical and optical properties of bulk and nanostructured materials.^2–5^ A special case is the single ion implantation regime, a condition where the distance between two neighboring ion tracks and their collision cascades is large enough to consider their interaction negligible.^6^ In the high energy regime, where electronic stopping prevails, cylindrically shaped disordered or amorphous regions of a few nm in diameter are often observed, *e.g.*, by transmission electron microscopy (TEM).^7–9^ These ion tracks have been used to nanostructure materials,^4^ fabricate microfilters and nanopore templates,^10^ and are important for the study of radiation resistance of materials.^2,11,12^ However, single ion tracks formed by ions with an energy where the nuclear stopping prevails are less studied, and typically using indirect methods,^13^ although some studies in, *e.g.*, crystalline Si exists.^7^ Further, materials with high tolerance to radiation are crucial for space applications, so the development of methodologies to study the physical properties of single ion tracks is of great importance and a big challenge.

ZnO presents excellent properties for optoelectronic applications due to its wide band gap energy of 3.37 eV at room temperature (RT), large free exciton (FX) binding energy (60 meV) and high luminescence efficiency.^14^ It is suitable candidate for solar cell applications,^15^ blue/UV light emitting diodes^16^ and lasers,^17,18^ UV photo detectors^19^ or transparent thin film transistors.^20^ Unintentional impurities like H, In, Al or Ge could be present in pristine ZnO as result of the crystal growth process. In fact, pure ZnO present naturally n-type conductivity with carrier concentration of ∼10^14^ to 10^17^ cm^−3^ due to native and unintentional donor impurities.^14,21^

ZnO reportedly has an extraordinary high radiation tolerance in comparison to other semiconducting materials such as Si, GaN or GaAs.^8,12,22^ Up to now, however, single ion impacts in ZnO have not been observed due to their nanoscale dimensions, neither the effects of ion tracks on the optical properties and the role played by impurities have been addressed. In this work, ion implantation effects have been studied in high quality hydrothermally grown ZnO single crystals. The germanium (Ge) ions were implanted with high kinetic energies (MeV) and low doses ranging from 5 × 10^7^ to 5 × 10^9^ cm^−2^ that resulted in damaged regions of 400–800 nm in depth. The as-implanted samples were characterized optically by combining depth-resolved cathodoluminescence (DR-CL) and photoluminescence (PL) spectroscopy. The low-dose ion implantation allowed for studying of single ion impacts and exposing different defect distributions through the analysis of combined hyperspectral imaging and DR-CL results.

## Experimental

A hydrothermally grown (0001)-oriented ZnO single crystal (obtained from Tokyo Denpa) was cut in six pieces of 5 × 5 mm^2^ using a laser cutter. The samples were subsequently cleaned for 5 minutes in ethanol, isopropanol and de-ionized water using an ultrasonic bath. One sample was kept as reference of pristine ZnO (labeled as Ref.) and four samples (Zn-face oriented) were then implanted with Ge ions in a NEC tandem ion implanter with energy of 1.85 MeV up to a dose of 5 × 10^7^, 1 × 10^8^, 1 × 10^9^ or 5 × 10^9^ cm^−2^, labeled from 5E7 up to 5E9, respectively. Additionally, one sample was implanted with 1.00 MeV Ge^+^ to a dose 1 × 10^9^ cm^−2^. No thermal annealing was performed after ion implantation to preserve the original network of ion tracks and damage in the samples.

The CL measurements were performed with a Delmic SPARC-system mounted on a JEOL JSM-IT300 scanning electron microscope (SEM). An Andor Shamrock SR-193i spectrometer with a 500 l mm^−1^ grating and a charged couple device (CCD) Andor Newton DU940P-BU2 detector was used to collect the spectra. Acceleration voltages from 2 to 30 kV and probing currents (*I*_b_) from 10 pA to 1 nA were used for the experiments in order to control the CL probing depth and excitation power. CL measurements were performed at temperatures from 80 to 300 K, using a Gatan cooling stage with liquid nitrogen.

Prior to SEM characterization, the samples were cleaned simultaneously for 5 min by low energy oxygen–argon plasma (25% O_2_, 75% Ar) in a Fischione 1020 Plasma Cleaner to remove and minimize possible effects induced by residual contamination on the surface.

The PL measurements were carried out at temperatures of 10 K and 80 K using a 325 nm wavelength cw-HeCd laser as excitation source. The emission was analyzed by a fiber-optic (Ocean Optics, HR4000) and imaging spectrometer systems (Horiba iHR320 coupled to Andor iXon888 EMCCD) with a spectral resolution below 0.2 nm in both cases.

**Fig. 1:**
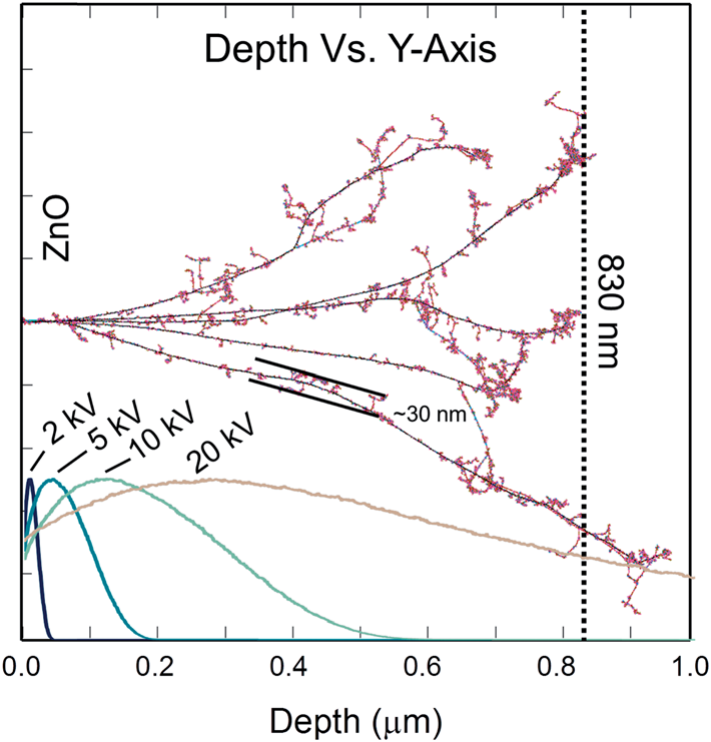
TRIM simulation of five collision cascades, using 1.85 MeV implanted Ge ions in ZnO. The vertical dotted line is the estimated projected range for the Ge-ions. The curves show the calculated electron energy loss distribution using CASINO for different acceleration voltage (*V*_acc_).

Fig. 1 shows the collision cascade of five single Ge ions trajectories in ZnO simulated by Monte Carlo based TRIM/SRIM software.^23^ Each cascade shows formation of point defects confined to a relative narrow region of about 30 nm diameter.^7^ According to the simulations, the projected range for 1.85 MeV Ge ions in ZnO is found to be 830 nm deep. In addition, Fig. 1 shows the calculated electron energy loss for the fast electrons forming the electron beam in the SEM as a function of depth for different acceleration voltages (*V*_acc_) simulated by the CASINO software.^24^ The most important energy loss mechanism within this energy range is excitation of electron hole pairs (ehp) and therefore the energy loss depth distribution can be approximated as the distribution of ehp-excitation. It is also worth noting that before the ehp recombine, they may both diffuse and drift away from the point of excitation. However, by varying the acceleration voltage of the fast electrons, ehp-excitation can be adjusted to occur predominately either close to the surface or within the implanted region, thus allowing for depth resolved (DR) CL spectra.

## Results and discussion

**Fig. 2:**
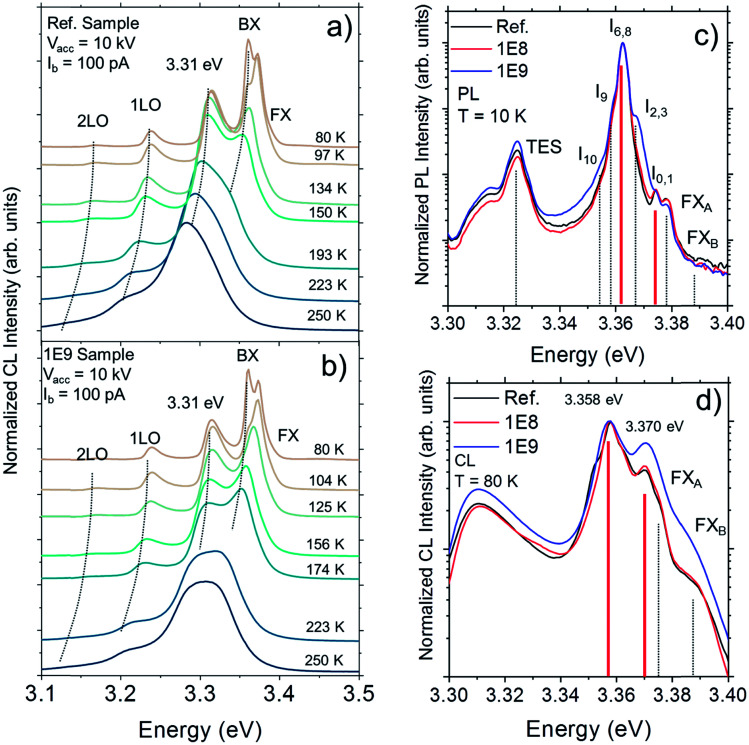
Temperature dependent CL of (a) un-implanted sample and (b) 1E9 sample. (c) PL spectra of un-implanted and implanted samples measured at 10 K. Vertical lines in (c) represent the reported positions of *I*-lines in ZnO. (d) Normalized CL intensity within the NBE measured at 80 K for the un-implanted and implanted samples measured at 4 kV. Solid lines in (d) correspond to *I*_6,8_ (D^0^X) and *I*_0,1_ (D^+^X) lines in (c) red-shifted by ∼5 meV to fit the peak maxima considering band gap narrowing at 80 K. Each CL-spectra were acquired from an area of 10 × 10 μm^2^.

Temperature dependent CL measured in the range from 80 to 250 K using an acceleration voltage of 10 kV reveals clear differences between the un-implanted and implanted (1E9) samples as can be observed in Fig. 2(a) and (b), which presents the normalized luminescence intensity in the near band edge (NBE) emission range from 3.1 to 3.5 eV. The spectrum for each temperature is shifted vertically for clarity. At 80 K, the CL-spectra show a band around 3.31 eV followed by longitudinal optical phonon replicas at lower energies (marked as 1LO and 2LO) separated by about 71 meV. The 3.31 eV peak is commonly attributed to the first longitudinal optical phonon replica of the free exciton (FX) emission (FX-1LO), but its actual interpretation has been subject of controversy and remains unclear.^25–28^ On the high energy side we can find the contributions from bound excitons (BX) and FX.^29^ The former dominate the spectrum while the latter can be observed as a weak shoulder that extends up to ∼3.39 eV. It can be noticed that the BX related peak emerge at temperatures below 150 K in the un-implanted sample [Fig. 2(a)], whereas it starts to emerge below 223 K in the implanted sample [Fig. 2(b)]. Indeed, BX peak clearly dominates the CL spectrum for the 1E9 sample at temperatures below 174 K. At 250 K, the details of the different transitions start to emerge. However, the NBE of the implanted (1E9) sample appears blue-shifted and broadened as compared to that of un-implanted sample. It is important to note that this can also be observed at room temperature and no heating was induced with the e-beam during the CL measurements (see Fig. S1 and S2†). Bearing in mind that the initial density and nature of both native defects and impurities before implantation are the same in the samples (all cut from the same wafer), the effect observed at the NBE luminescence after thermal quenching likely suggest a slight modification of the binding energy of the donor impurities in the vicinity of the ion tracks caused by local stress.^30^

The CL-measurements are limited to liquid N_2_ temperatures; hence to reveal more detailed structure of emission in the NBE-region, the complementary PL measurements were performed at 10 K. Fig. 2(c) shows normalized PL spectra for the implanted and un-implanted samples. At the low energy side of the spectrum, the 3.31 eV band is overlapping with the two-electron-satellite transition (TES) at ∼3.32 eV. At higher energies, the spectral positions of the different *I*-lines are indicated by a set of vertical markers in accordance with the reported energies in the literature.^21,29,31^ The predominant PL peak is located at 3.362 eV and fits well with the two closely positioned *I*_6_ and *I*_8_ lines typically assigned to a neutral donor bound excitons (D^0^X).^31^ One can observe that the line at 3.374 eV agrees well with the *I*_0_ and *I*_1_ lines associated with excitons bound to ionized donors (D^+^X). Recently, Heinhold *et al.*^21^ reviewed a variety of observable excitonic emissions and their excited-state transition in ZnO. The *I*_0_ and *I*_6_ lines were assigned to excitons bound to ionized and neutral Al_Zn_, respectively. Similarly, the *I*_1_ and *I*_8_ excitonic lines correspond to ionized and neutral Ga_Zn_ defects. Apart from the thermal NBE shift due to band gap narrowing (BGN),^32^ the energy separation of about 12 meV between neutral D^0^X and its ionized counterpart D^+^X observed at 10 K is not expected to change significantly upon the temperature increase up to 80 K (BGN is Δ*E*_g_ ∼ 5 meV).^33^ In Fig. 2(d), an enlargement of the spectral region from 3.30 to 3.40 eV of the CL spectra measured at 80 K from the un-implanted and implanted samples are shown alongside for comparison. It is worth mentioning that the CL spectrum at 80 K was acquired with *V*_acc_ = 4 kV, which corresponds to a probing depth similar to the penetration depth of a 325 nm laser excitation (α^−1^ ≤ 100 nm (ref. 34 and 35)) in ZnO (see Fig. S3†). The spectral features above the 3.31 eV peak are well resolved and dominate the luminescence spectrum with two peaks located at 3.358 eV and 3.370 eV. Interestingly, when comparing the normalized CL spectra, the relative intensity of 3.370 eV emission clearly increases as a function of dose. The high energy side of the NBE at low temperatures is generally attributed to excited states of bound excitons, excitons bound to ionized donors^29^ or surface excitons (SX).^36–39^ However, SXs are generally observed in samples with high aspect ratio, like microrods, nanowires^36,37,39^ and thin films,^38^ with the peak position reportedly around 3.364–3.367 eV at ∼10 K.^38,39^ In the present study, the PL spectra measured at 10 K show a peak at 3.374 eV. Therefore, it is reasonable to assume from the comparison that the band at 3.370 eV measured at 80 K originates from the recombination of excitons bound to ionized donors (Al_Zn_^+^ or Ga_Zn_^+^).

**Fig. 3:**
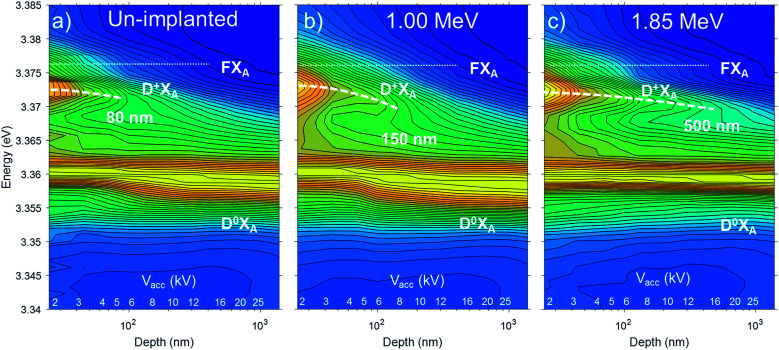
Normalized DR-CL maps recorded at 80 K for ZnO samples: (a) un-implanted, and implanted samples with fluence of 10^9^ cm^−2^ and ion energy of (b) 1.00 MeV and (c) 1.85 MeV.

The depth distributions of the implantation induced defects were investigated by DR-CL experiments performed at 80 K and constant excitation power while varying *V*_acc_ in the range from 2 to 25 kV (*i.e.* maintaining constant *I*_b_*V*_acc_). In these measurements the NBE of the un-implanted and two samples implanted with 1 × 10^9^ cm^−2^ Ge ions having energies 1.00 and 1.85 MeV, corresponding to ion ranges of ∼400 nm and ∼830 nm, respectively, are compared. Normalized DR-CL results are shown in Fig. 3(a)–(c) in the form of CL intensity maps as a function of the acceleration voltage *V*_acc_. The approximate probing depth, defined as the mean position of the electron energy loss distribution estimated by CASINO simulations, is plotted along the *X*-axis.

The dotted lines at the top of Fig. 3(a)–(c) mark the position of FX emission that also appears as a weak shoulder in Fig. 2(d). In the un-implanted sample in Fig. 3(a) for *V*_acc_ < 4–5 kV, the peak originating from the D^+^X is clearly observed and similar in intensity as the corresponding neutral D^0^X. Interestingly, as *V*_acc_ is increased (*i.e.*, for greater probing depth), the relative D^+^X intensity is quickly reduced. Since the generated electron hole pairs can diffuse (the diffusion length in bulk ZnO is ∼100–200 nm at 80 K)^40^ and drift towards the surface, one cannot based on this data alone conclude whether the increased relative intensity of the D^+^X line is caused by the surface or sub-surface damage (*e.g.*, introduced by sample polishing),^41^ or by the presence of surface band-bending.^25,42,43^ The main point, however, is that in the samples implanted up to a dose of 1 × 10^9^ cm^−2^ with energy of 1.00 and 1.85 MeV, the shift reaches deeper into the sample, which is consistent with the deeper projected range of implantation of ∼400 nm and ∼830 nm, respectively.

**Fig. 4:**
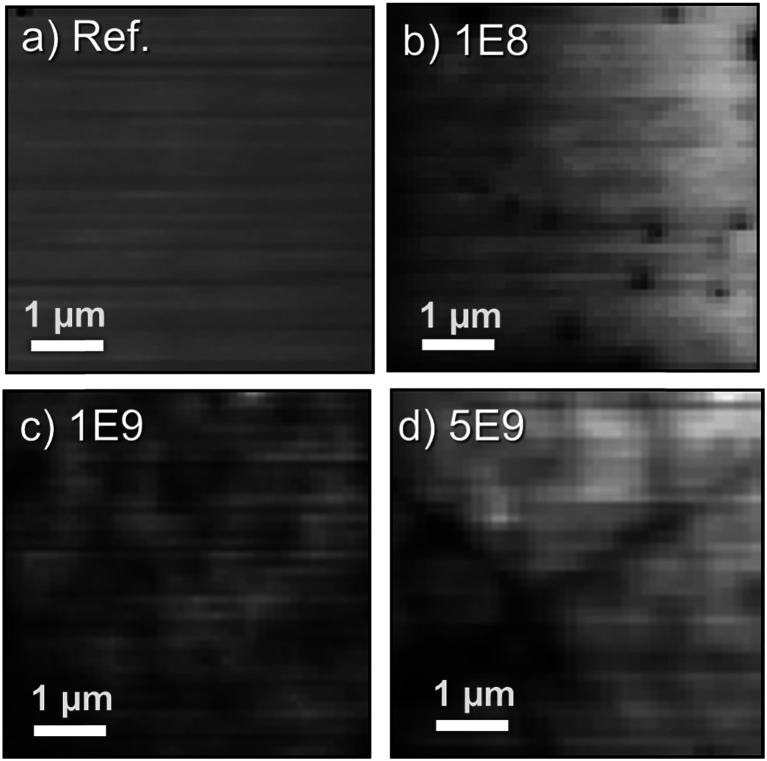
Total CL Intensity maps measured at 80 K of (a) un-implanted reference, (b) 1E8, (c) 1E9, and (d) 5E9 samples.

Hyperspectral CL imaging has been performed at 80 K to analyze the effect of the ion bombardment on the local luminescence properties of the implanted ZnO samples. The CL maps shown in Fig. 4 display the total CL intensity recorded on areas of 5 × 5 μm^2^. To achieve optimal tradeoff between probing depth and spatial resolution (100 nm per pixel), the maps were acquired with e-beam energy of 5 keV and with a 20 pA current, similar to that reported by Ruane *et al.*^44^ Horizontal lines on the CL maps are artefacts likely caused by charging effects during scanning and small fluctuations of the e-beam current.

In the CL map of the un-implanted sample [Fig. 4(a)] there are no regions with significant variation in the CL intensity. By contrast, the implanted samples depicted in Fig. 4(b)–(d) clearly show regions with reduced emissions that become more pronounced as the ion dose increases. In the CL map of the sample 1E8 [Fig. 4(b)], the distinct isolated dark spots are identified as the signature of single ion impacts. Here, it should be noted that a dose of 1 × 10^8^ cm^−2^ corresponds to one ion track per μm^2^. However, as displayed in Fig. 1, the defect generation from a single ion is highly non-uniform in the secondary collision cascades. Hence, with a probing depth of ∼100–200 nm, not all the ion tracks will be visible. Further, a lateral resolution of 100 nm per pixel makes the identification of single ion impacts difficult in 1E9 and 5E9 samples presented in Fig. 4(c) and (d), respectively. However, dark and bright regions due to random distribution of the implanted ions can still be perceived. Additionally, the sample 5E9 is characterized not only by random changes in the brightness, but also extended dark regions indicating defects of different nature, like dislocations or stacking faults.^27^

**Fig. 5:**
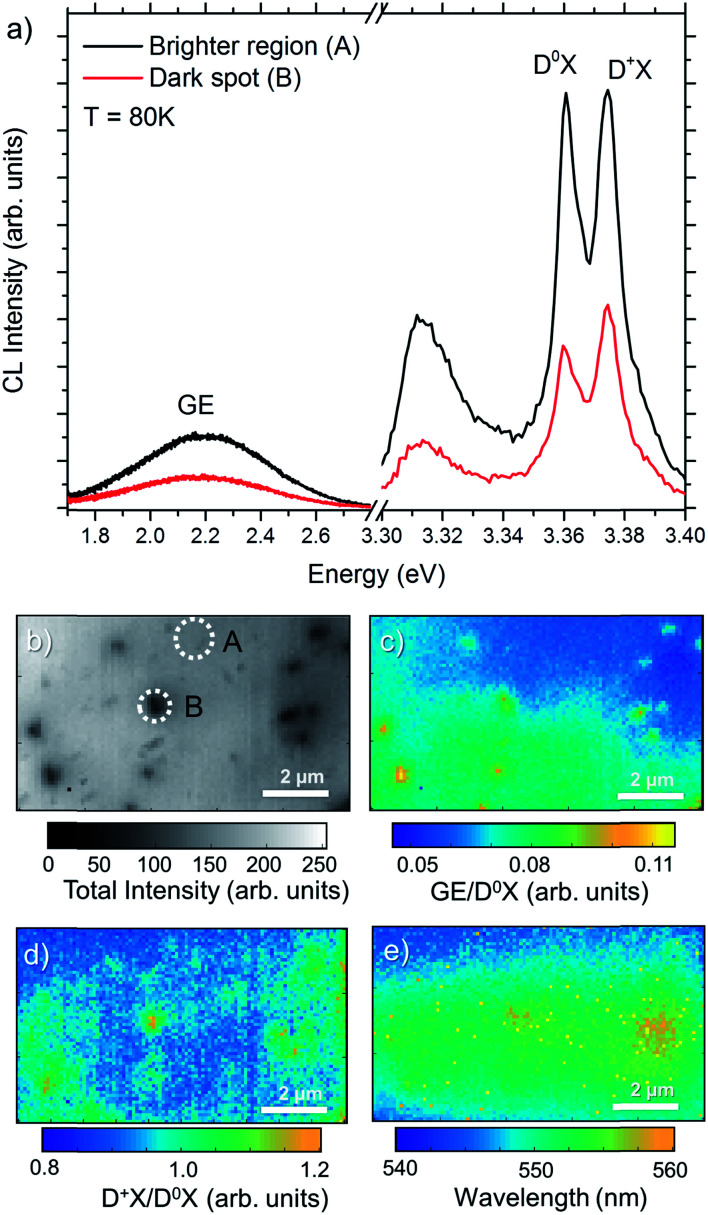
(a) Green emission (GE) and near band edge (NBE) regions of the CL spectra measured at the center of an ion track (dark spot) and undamaged area (brighter region). (b) Total CL Intensity map measured at 80 K of 5E7 sample and its corresponding (c) [GE/D^0^X] map, (d) [D^+^X/D^0^X] map and (e) central wavelength map of the GE band. Spectra in (a) correspond to A and B regions marked in (b).

Fig. 5(a) shows the CL spectra measured at the center of a dark spot identified as an ion track, and on an undamaged area for the sample 5E7, from A and B areas marked in Fig. 5(b). It is important to note that the measurements within the ion-track also include luminescence from a larger area surrounding the track, *i.e.*, at least some of the D^0^X signal comes from an undamaged part of the sample. In addition to D^0^X and D^+^X bands at the NBE, the CL spectra show the broader and weaker emission in the visible region peaking at around 2.2 eV, which is attributed to several different deep defect levels, but commonly referred to in general as green band emission (GE).^14,20,45^ In the case of 5E7 sample, the average distance between the adjacent ion implants is expected to be greater than 1 μm. Fig. 5(b) represents the total CL intensity map of a 10 × 6 μm^2^ area, and hence, will in average contain ∼30 ion tracks. In the figure, several dark spots can be identified. However, the intensity decrease and extent of the dark region varies significantly. Indeed, only a few identified dark regions (∼10) show a strong intensity contrast and these will be discussed further. Importantly, this is in accordance with the implanted dose and SRIM calculations demonstrating a highly non uniform defect generation, as discussed above, where only a fraction of the ion tracks exhibit a pronounced defect generation within the probing volume (100–200 nm in depth). The CL intensity in the center of the dark spots is decreased by 50–60% over the full spectrum, as observed in Fig. 5(a), with respect to the surrounding areas, indicating that the generated carriers recombine non-radiatively near the ion track core. Interestingly, since every pixel contains full spectral information, additional analysis can be performed by calculating the intensity ratio of different luminescence bands, *e.g.*, [GE/D^0^X] and [D^+^X/D^0^X], as shown in Fig. 5(c) and (d), respectively. These maps represents the local GE or D^+^X peak intensity normalized with respect to the excitonic D^0^X emission peak [marked in Fig. 5(a)], allowing for considerable increase of the contrast compared to the total intensity map of Fig. 5(b). The [GE/D^0^X] map indicates the preferential luminescence mechanism, favoring the recombination through deep defect levels formed by the ion tracks. The [D^+^X/D^0^X] map [Fig. 5(d)] also shows inhomogeneous distribution over the scanned area. In general, dark spots in Fig. 5(b) show a slightly higher [D^+^X/D^0^X] ratio (yellowish areas) indicating that the luminescence from D^+^X increases upon D^0^X at the ion track region. Finally, Gaussian fitting on hyperspectral data has been performed to estimate the central wavelength of the GE band. The resulting wavelength map is shown in Fig. 5(e). Bright/yellow pixels are artefacts caused by failed curve fitting. Despite the fact that the features are not as sharp as compared to the [GE/D^0^X] ratio map, the spatial distribution of the central wavelength of the GE band show a slight redshift (few tens of meV) in the areas exhibiting presence of ion tracks.

**Fig. 6:**
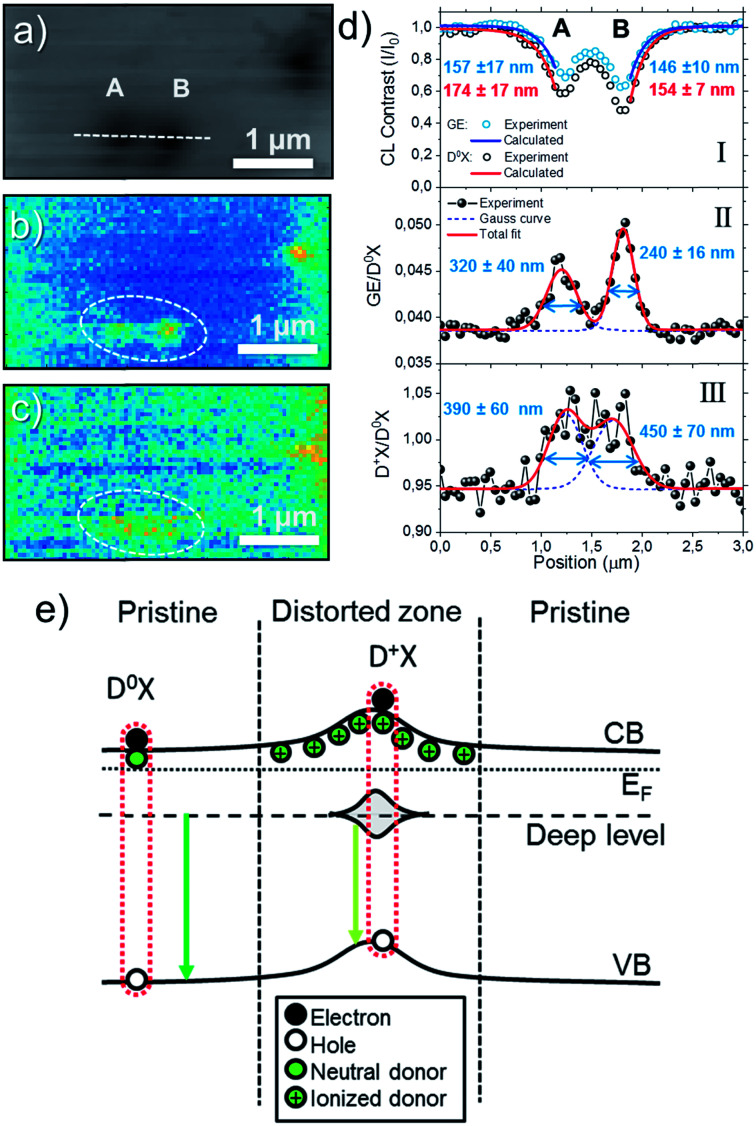
(a) Total CL Intensity maps measured at 80 K of the 5E7 sample and its corresponding (b) [GE/D^0^X] and (c) [D^+^X/D^0^X] maps. (d) Intensity profiles acquired from the linescan marked by the dashed line in (a) corresponding to: (I) CL contrast from D^0^X and GE, (II) [GE/D^0^X] and (III) [D^+^X/D^0^X]. (e) Band diagram schema for a single ion track.

Additional information has been obtained from the analysis of the Hyperspectral CL data. Fig. 6(a) shows the total CL intensity map measured at 80 K of an area of 4 × 2.5 μm^2^ with 50 nm per pixel resolution where three ion impacts are observed. As seen in Fig. 6(a), the distance between the implanted ions is around 600 nm. Fig. 6(b) and (c) correspond to the [GE/D^0^X] and [D^+^X/D^0^X] ratio maps, respectively. Unlike [D^+^X/D^0^X] map, stronger contrast and sharper features are obtained by mapping the [GE/D^0^X] ratio. Fig. 6(d) shows the line-scans across two close ion impacts (labeled as A and B) in Fig. 6(a). Panel I shows CL contrast profiles from the D^0^X and GE bands defined by *I*_CL_(*x*)/*I*_CL,0_, where *I*_CL,0_ is the intensity of the luminescence bands measured in a pristine region. For comparison, the corresponding line-scans from the [GE/D^0^X] and [D^+^X/D^0^X] maps are shown in the panels II and III of the Fig. 6(d), respectively.

In general, the interpretation of CL results is complicated and multiple factors influence the CL intensity in a locally distorted and defective system. The CL signal (*I*_CL_) involves carrier generation, diffusion and recombination processes, which is given by *I*_CL_ = ∫*p*(*r*)/*τ*_*r*_d*V*,^46^ where *p*(*r*) is the excess of carriers generated by the e-beam and *τ*_*r*_ the lifetime considering the radiative recombination. The minority carrier diffusion length *L* is related to the carrier lifetime by the formula 
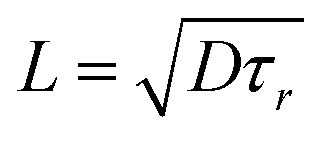
 where *D* is the carrier diffusivity. Since ion tracks introduce non-radiative recombination channels along a cylinder-like column, the situation can be equated to non-radiative threading dislocations, where CL contrast profile analysis has been used to study the diffusion length and carrier lifetimes in materials such as GaN, GaAs and ZnO.^46–50^ In a simple case where the carrier generation distribution is considered constant, an approximation of the luminescence intensity using the analytical solution for the carriers diffusion equation is *I*_CL_(*x*)/*I*_CL,0_ ∝ 1 − exp(−*x*/*L*),^50^ which is valid for defective regions with radius *r*_0_≪*L* and e-beam positions *x* relatively far from the defect core. The calculated diffusion length from D^0^X and GE using the exponential approximation are around 150–170 nm. Even though these numbers should be considered as estimations, the calculated *L* values are close to the expected diffusion length for ZnO, which is of the order of 100–200 nm at 80 K.^40^ It can be observed from Fig. 6(d)-I, that the calculated *L* are slightly higher in the ion track A compared to B, contrary to the luminescence decrease, indicating that the region represent the influence of the local distortion on the optical emission, *i.e.* the extent of the ion track, and not given by the resolution of the system. It should be noted that an important difference between the present observation and that of, *e.g.*, dislocations that may also be present in the material, is that the density of point defects is higher and the effective radius of the defective region surrounding the ion track depends on the amount of energy loss by the implanted ion on each collision during its trajectory. Thus, while CL contrast profiles might vary from one ion track to another, dislocations should look similar.

Despite the fact that the lower luminescence efficiency is caused by non-radiative recombination channels, the relative intensity from the GE profile observed in Fig. 6(d)-I is higher compared to the D^0^X profile as the e-beam approaches the ion track core, which can be interpreted as a higher ratio of radiative recombination events by defects. Since the ion impacts mainly create intrinsic defects and the projected range of the ion is outside the probing region, we will assume in the following that the [GE/D^0^X] contrast is caused by intrinsic defects. Further, the [GE/D^0^X] profile shown in Fig. 6(d)-II is sharper compared to the intensity contrast *I*_CL_(*x*)/*I*_CL,0_ shown in panel I, with an increase of 25–30% and recovering the bulk value near half of the distance between the ion tracks A and B. Hence, the intensity ratio maps are more effective to estimate defect distributions in comparison to the intensity profile of a single luminescence band. In this case, for simplicity, the defect profiles have been fitted by Gaussian curves. The radius of the defective region can be estimated by half of the calculated full width at half maximum (FWHM) resulting in 160 ± 20 and 120 ± 8 nm for the ion tracks A and B, respectively. Importantly, the calculated radii are greater than the dimensions of a single ion track formed by swift ions where the electronic stopping prevails, as, *e.g.*, seen by TEM >100 MeV ions.^9^

The profile acquired from the [D^+^X/D^0^X] map [Fig. 6(d)-III] show the increase of ionized donor bound excitons with respect to the neutral ones, which is of the order of 10% near the center of the ion tracks. It can be noted that from Fig. 5(c) not all ion tracks show significant increase of [D^+^X/D^0^X], which can be due to fewer defects near the surface, as previously discussed. In combination with the DR-CL analysis, the results indicate that the surface band bending and or defects-states can be mapped in the sub-micrometer scale using the local increase of D^+^X emission as tracer. Because of that, an estimation of the surrounding space charge radius calculated from the profiles shown in the Fig. 6(d)-III is ∼200 nm for both ion tracks.

The model represented in the Fig. 6(e) summarizes the main observations according to the CL analysis of single ion tracks. At low temperature (*e.g.*, 80 K), the luminescence from the pristine region is dominated by D^0^X excitons with a small portion of GE from deep defects levels randomly distributed in the material. In the ion track region, the non-radiative recombination increases due to structural distortions and point defects. At the same time, the higher density of point defects leads to increase of the GE emission with respect to DX. The deep band emission is of a vibronic nature, which includes a large amount of defect relaxation as a response to the change in charge state.^51,52^ The exact energy of the emission is highly sensitive to this relaxation, and it is speculated that the slight red-shift observed in Fig. 5(e) is caused by a change of the relaxation energy due to the high local concentration of defects. The observation of a higher [D^+^X/D^0^X] ratio in the implanted samples can be interpreted by the introduction of compensating defects, either as isolated acceptors or through the formation of donor–vacancy complexes (D_Zn_–V_Zn_),^53,54^ which will lower the Fermi-level and subsequently lead to ionization of the initially neutral donor (Al_Zn_ or Ga_Zn_) at 80 K, leading to the characteristic luminescence band identified at 3.370 eV in the CL spectra.

**Fig. 7:**
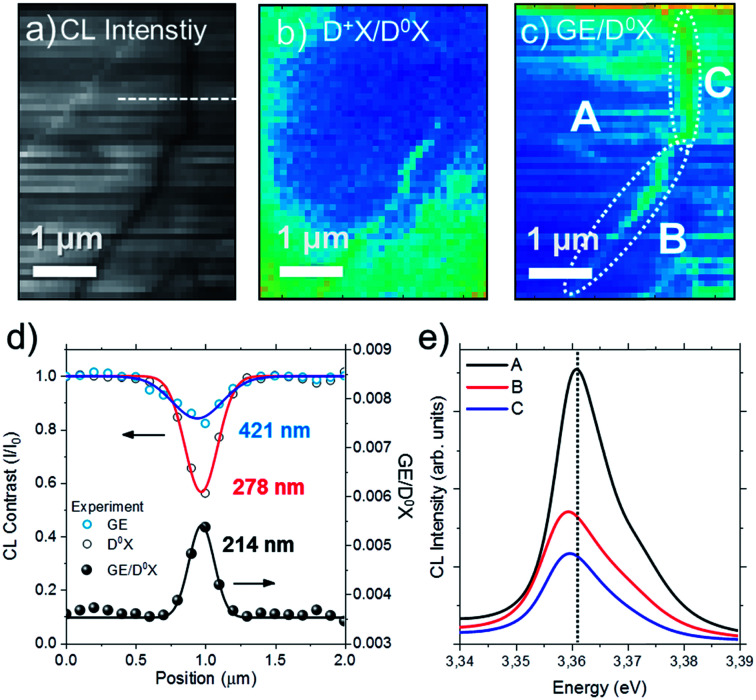
(a) Total CL intensity map at 80 K of the 5E9 sample and the corresponding (b) [D^+^X/D^0^X] and (c) [GE/D^0^X] ratio maps. (d) CL contrast and [GE/D^0^X] ratio profiles along the dashed line indicated in the panel (a). (e) NBE spectra from the region A and sections B and C marked in (c).

The situation is different if the sample is implanted with a higher dose. Fig. 7(a) shows the total CL intensity map measured on the 5E9 sample at 80 K and 5 keV with a long defect observed as a dark line with a clear trajectory that follow the crystalline directions in ZnO. Hyperspectral analysis of the [D^+^X/D^0^X] [Fig. 7(b)] and [GE/D^0^X] [Fig. 7(c)] maps reveal different characteristics compared to those observed for the single ion impacts. In Fig. 7(c) different areas and sections have been highlighted as A (outside of the extended defect), B and C. The [D^+^X/D^0^X] ratio is higher along the section B, while [GE/D^0^X] ratio is higher along both sections B and C. The D^0^X and GE contrast profiles along the dashed line in Fig. 7(a) are shown in Fig. 7(d) as well as the [GE/D^0^X] ratio. The profiles have been fitted by Gaussian curves. The main difference with respect to the single ion track regime is that in this case the appearance of non-radiative recombination near the defect clearly affects the D^0^X emission in a greater extent compared to the GE. Moreover, the FWHM of the Gaussian curve calculated from the [GE/D^0^X] profile [bottom profile in Fig. 7(d)] is 214 nm. The profile is narrower compared to the 240 and 320 nm calculated from the ion tracks in Fig. 6(d). Finally, the NBE emissions presented in Fig. 7(e) demonstrate a dominating peak at 3.361 eV in region A (marked by a dotted line), which appears red-shifted down to 3.360 eV in the regions B and C. It is also noteworthy that only a minor increase of emission at the high energy side attributed to D^+^X is noticeable in the hyperspectral analysis.

Usually, it is expected that dynamic annealing effects appear during implantation.^55^ Indeed, the minimum distance between two implanted ions in the 5E9 sample, considering a random distribution in a specific area, is about 50 nm, which means that defect regions and strained areas^9^ may overlap. Under these conditions, the formation of single ion tracks may be reduced and defect migration can be promoted, therefore, optical properties should be different compared to single ion track regime. High densities of point defects accumulate along dislocations thus compensating the lattice strain in the crystal as observed from Fig. 7(d). Finally, the peak shift observed at the defect region [Fig. 7(e)] can be due to the bandgap *E*_g_ shift induced by local strain fields from edge or mixed type dislocations.^49^

## Conclusions

We demonstrate that a single ion track induced changes of materials properties can be addressed on a nanoscale by combining depth-resolved hyperspectral CL and PL approaches. In particular, optical properties of single ion tracks have been studied in Ge implanted ZnO single crystals. Both CL and PL results indicate that ZnO is sensitive to implantation doses as low as 10^8^ to 10^9^ cm^−2^. The depth range of the damaged region has been identified by DR-CL using the D^+^X emission as a tracer of the local band bending. Regions with characteristic luminescence signatures that could be attributed to the presence of single ion tracks have been studied by hyperspectral CL imaging at 80 K with spatial resolution of 50 nm. CL contrast profile analysis across single ion tracks has been used to estimate the carrier diffusion length (around 150 to 170 nm) while [GE/D^0^X] and [D^+^X/D^0^X] ratio maps were used to estimate the range of defect radius (120 to 160 nm) and the space charge surrounding single ion tracks (around 200 nm). A model of upward band bending across a single ion track has been proposed by combining the results of spatially resolved CL mapping and spectral analysis. For 5 × 10^9^ cm^−2^ ion dose no signatures from single ion tracks were identified, instead defect aggregation along expanded defect was observed indicating that the single ion track regime has been exceeded.

## Conflicts of interest

There are no conflicts to declare.

## Supplementary Material

NA-002-C9NA00677J-s001
