# Supplementary material for: Optical signatures of single ion tracks in ZnO
Source: Nanoscale Adv. 2019 Dec 23;2(2):724–33. doi: 10.1039/c9na00677j (PMC9418517; doi:10.1039/c9na00677j)
Supplement: NA-002-C9NA00677J-s001 [file NA-002-C9NA00677J-s001.pdf]

Electronic Supplementary Material (ESI) for Nanoscale Advances.
This journal is © The Royal Society of Chemistry 2019

Supplementary Information

Optical signatures of single ion tracks in ZnO

G. C. Vásquez*, K. M. Johansen, A. Galeckas, L. Vines, B. G. Svensson

*Corresponding author
G. Cristian Vasquez
E-mail: g.c.vasquez@smn.uio.no
Centre for Materials Science and Nanotechnology, University of Oslo, N-0318 Oslo, Norway

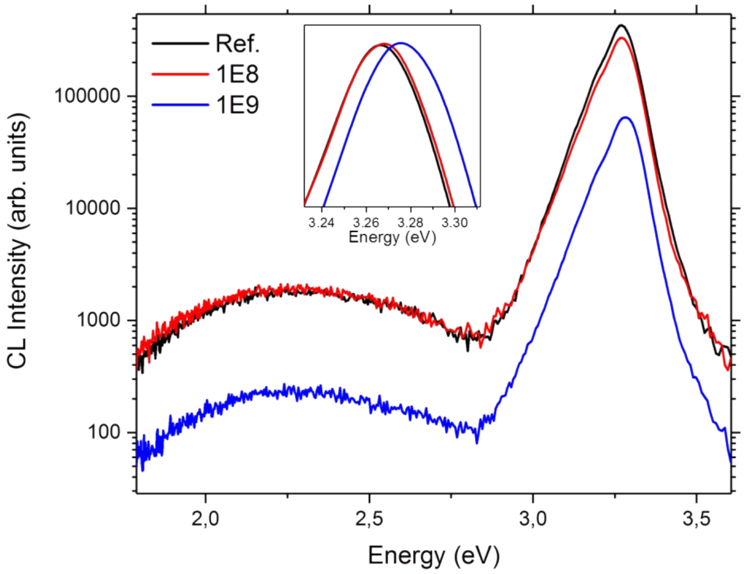

Figure S1. Room temperature CL spectra of ZnO samples acquired with V_{acc} = 5 kV and I_b = 50 pA. The inset shows a magnification of the NBE peak region for normalized spectra.

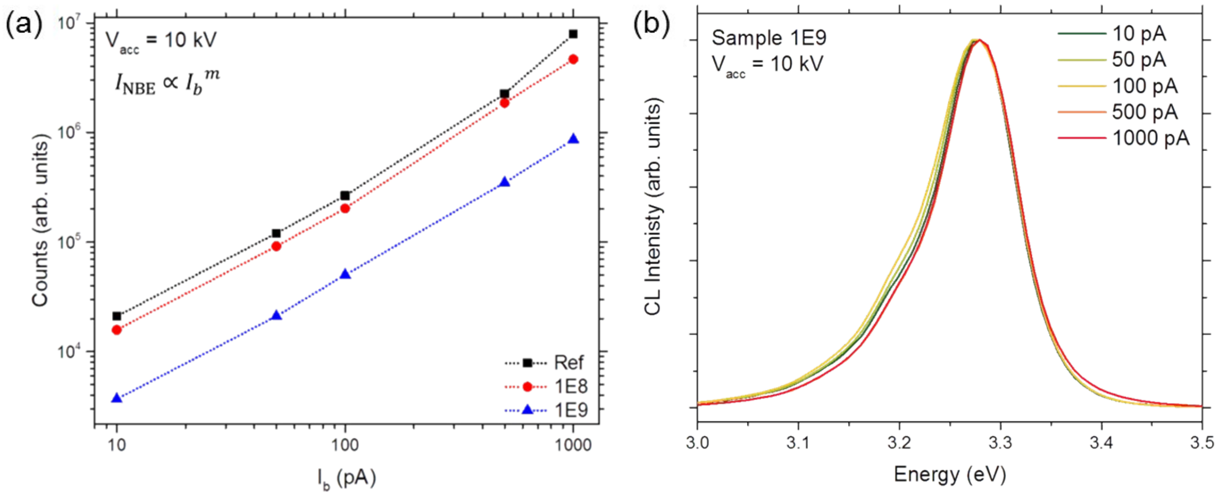

Figure S2. (a) NBE maximum intensity as a function of e-beam curren (I_b) and constant e-beam voltage (V_{acc}) and (b) the corresponding Normalized NBE spectra from sample 1E9.

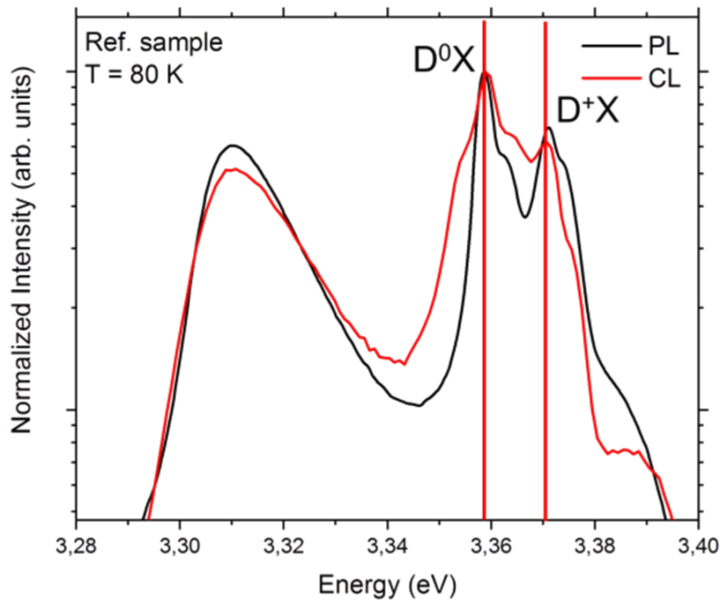

Figure S3. Normalized CL and PL spectra of the unimplanted sample measured at 80 K. Vertical lines represent the position of for $I_{6,8}$ (D^0X) and $I_{0,1}$ (D^+X) at low temperature (<10K) shifted ~5 meV to fit the peak maxima.

Figure S1 shows the CL spectra measured at room temperature (RT) on 10x10 μm^2 areas. Significant NBE blue-shifting can be noticed for 1E9 sample (inset). Power dependent-CL study was performed at RT to analyze possible thermal induced effects (e.g. band gap broadening and shifting) on the NBE peak intensity [Figure S2(a)] and spectral shape [Figure S2(b)]. We did not observe such effects using e-beam currents (I_b) up to 1 nA. The NBE peak intensity in Figure S2(a) can be approximated by a power law $I_{NBE} \propto I_b^m$ with m ~ 1, characteristic for free- and bound-exciton emissions.[1] On the other hand, the spectral shape is not affected significantly, so thermal effect can be neglected in the range of I_b used (20 to 100 pA). Figure S3 shows a comparison between CL and PL spectra at 80. The CL spectrum acquired at 4 keV shows similar characteristics as the PL spectrum.

1 A. Hamdouni, N. Ben Sedrine, J. C. Harmand and R. Chtourou, *Eur. Phys. J. Appl. Phys.*, 2007, **38**, 221–225.
